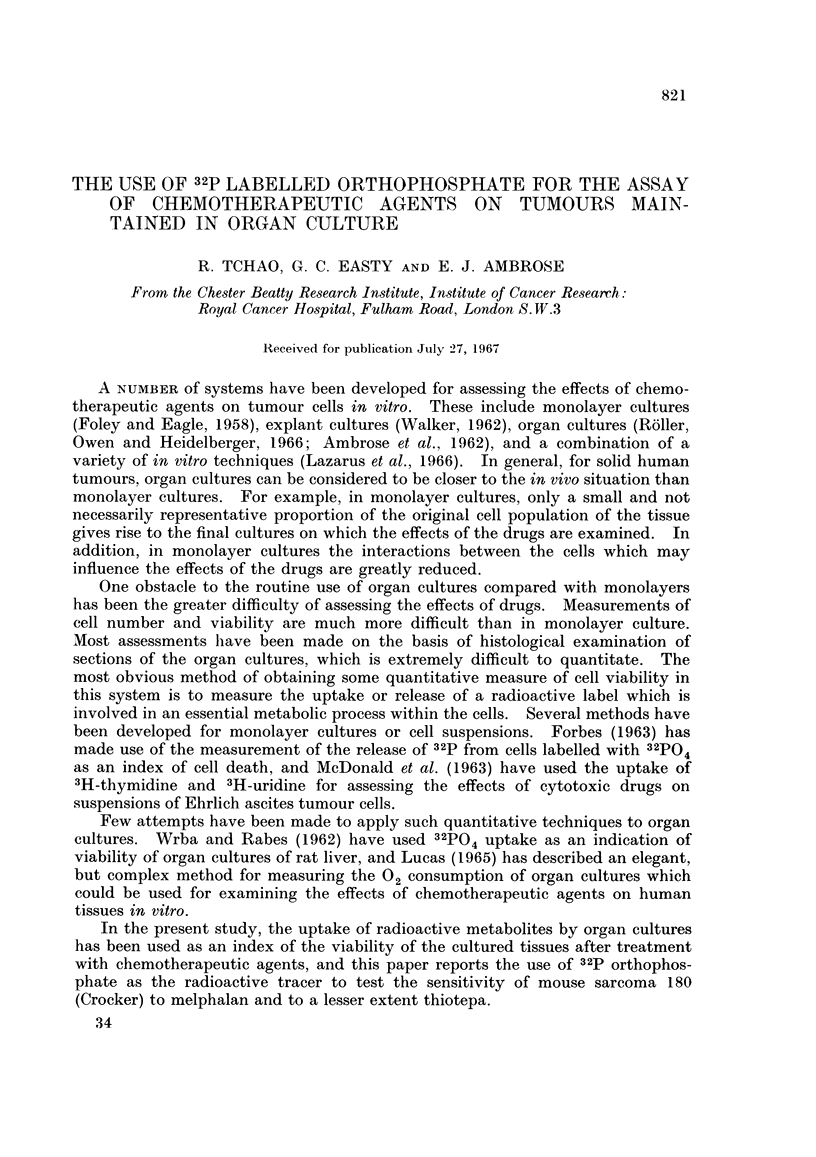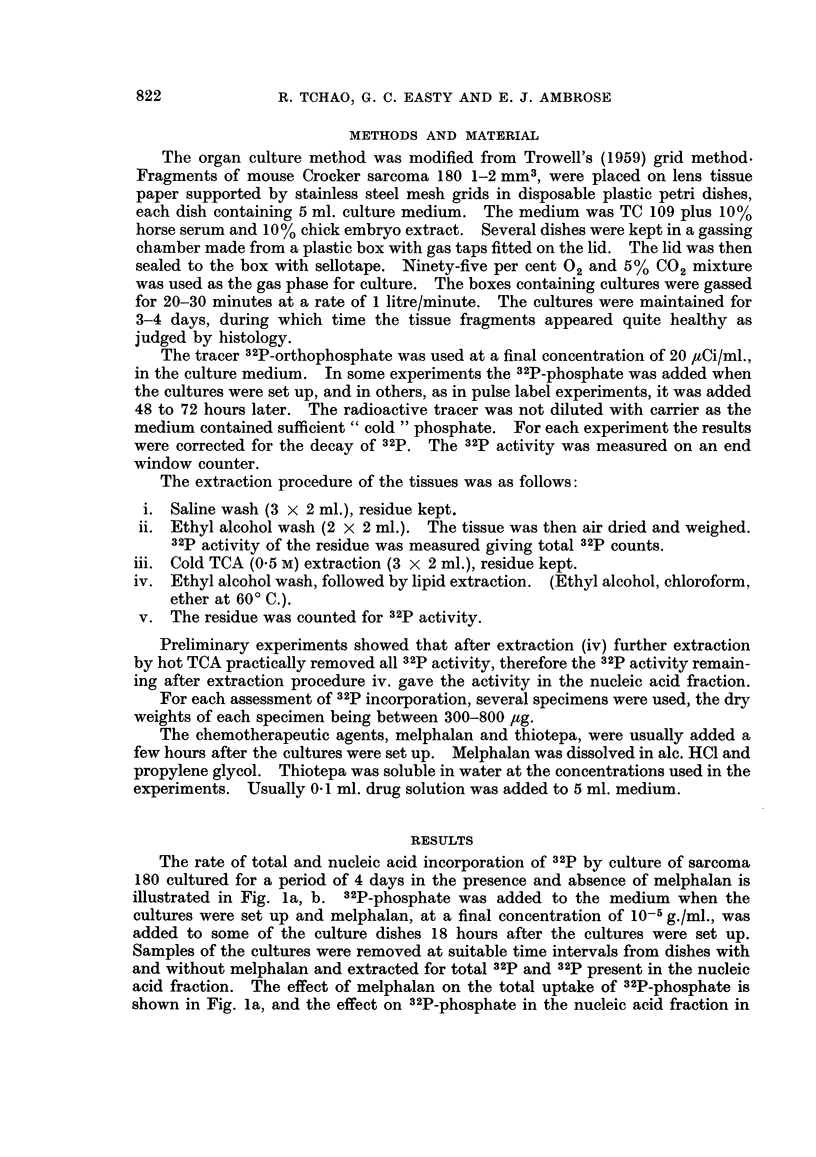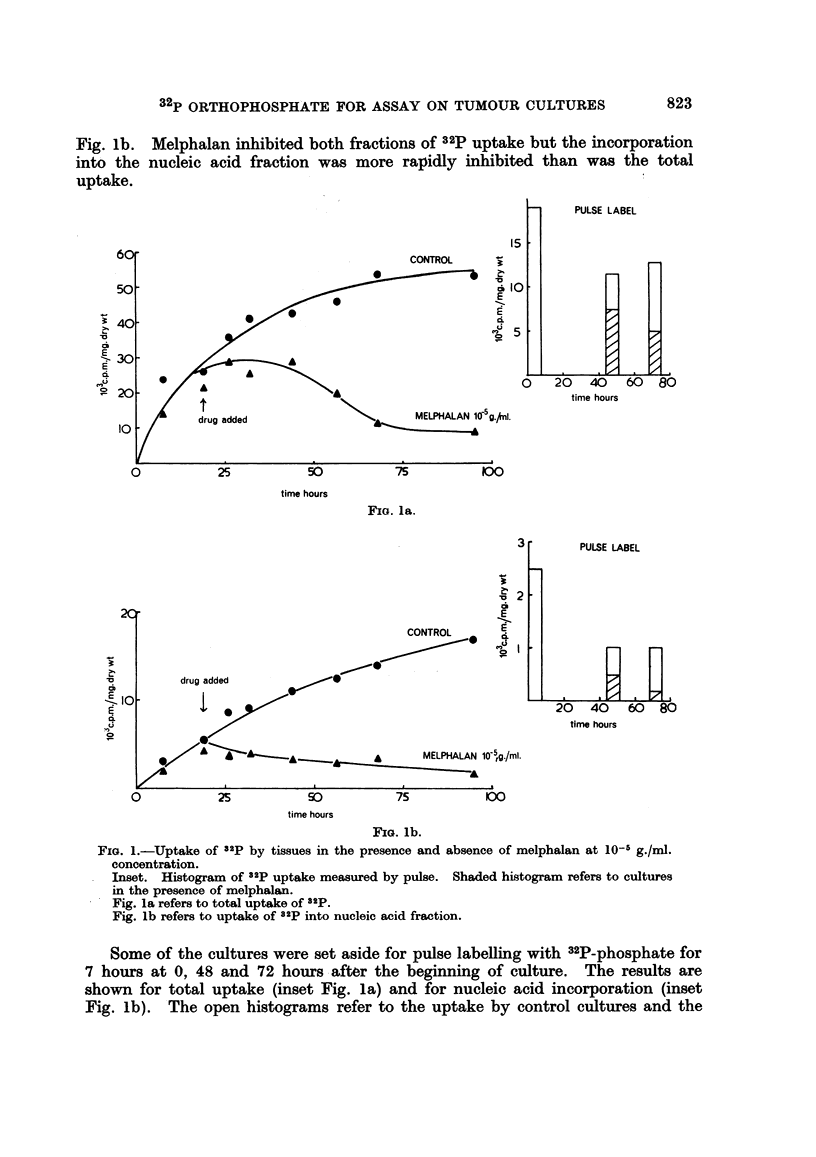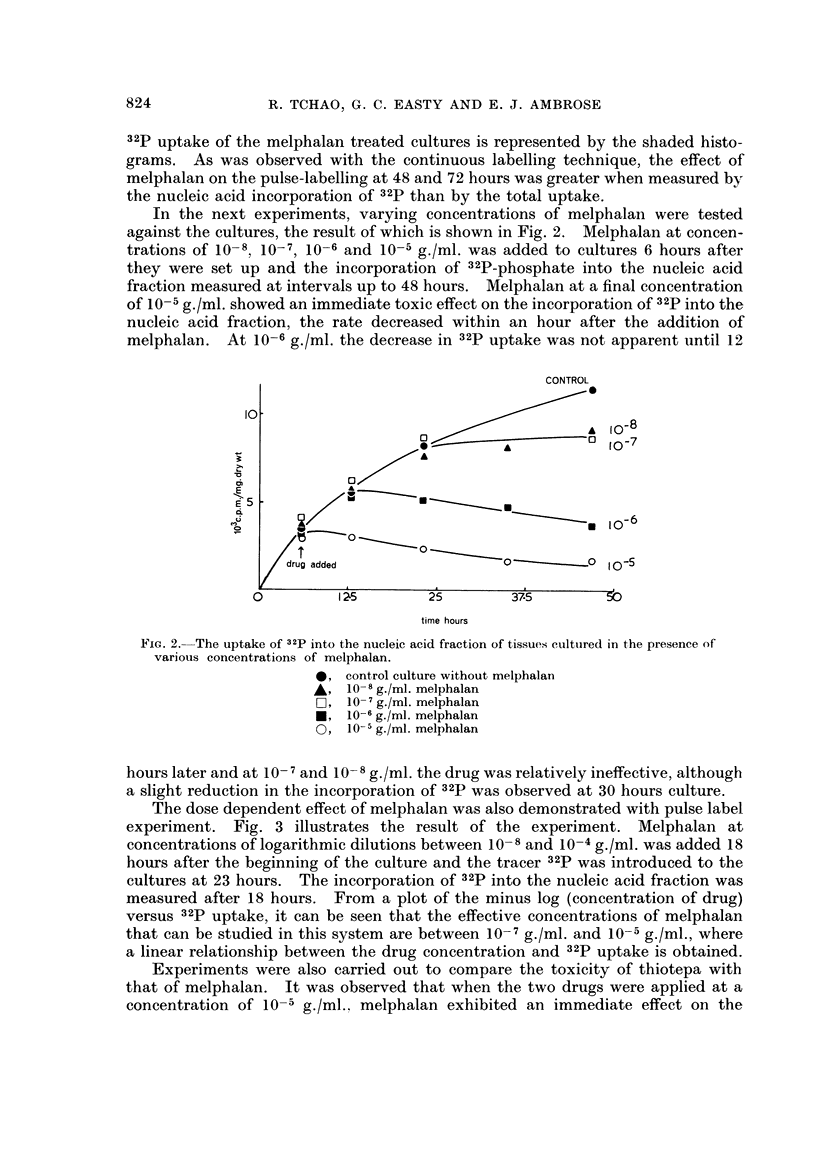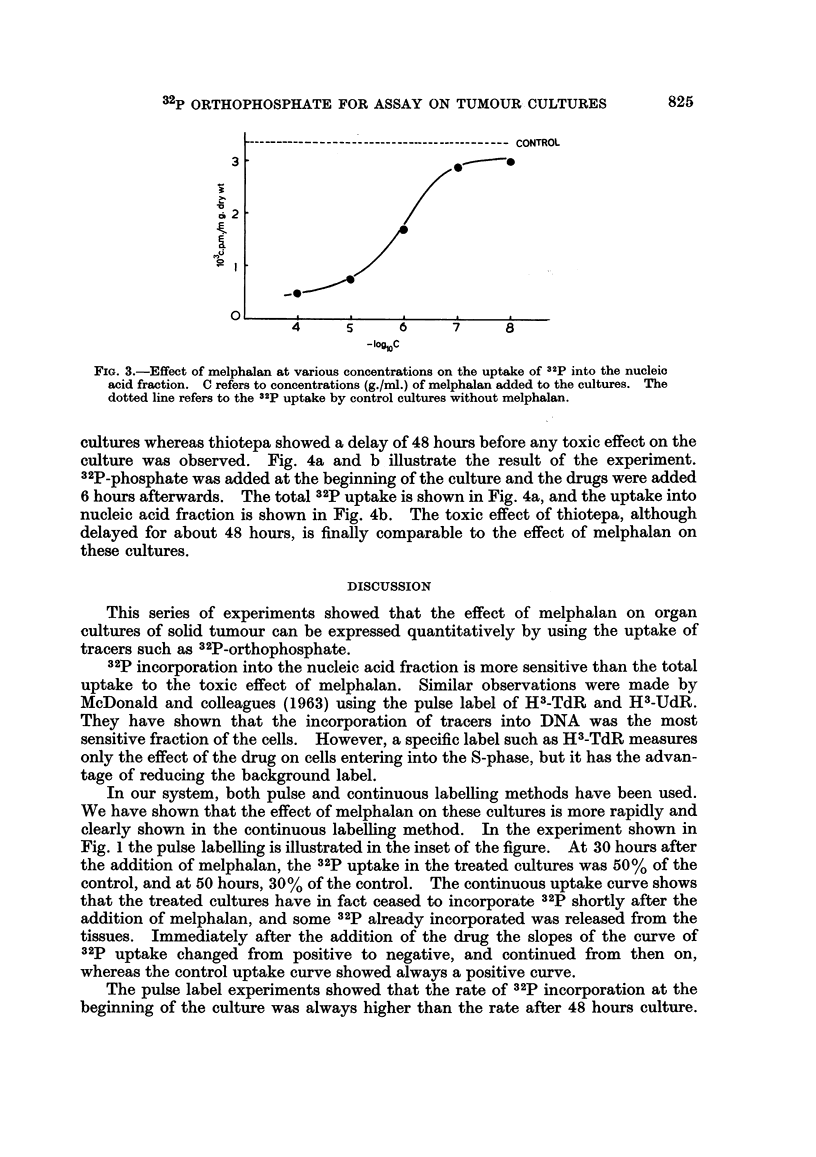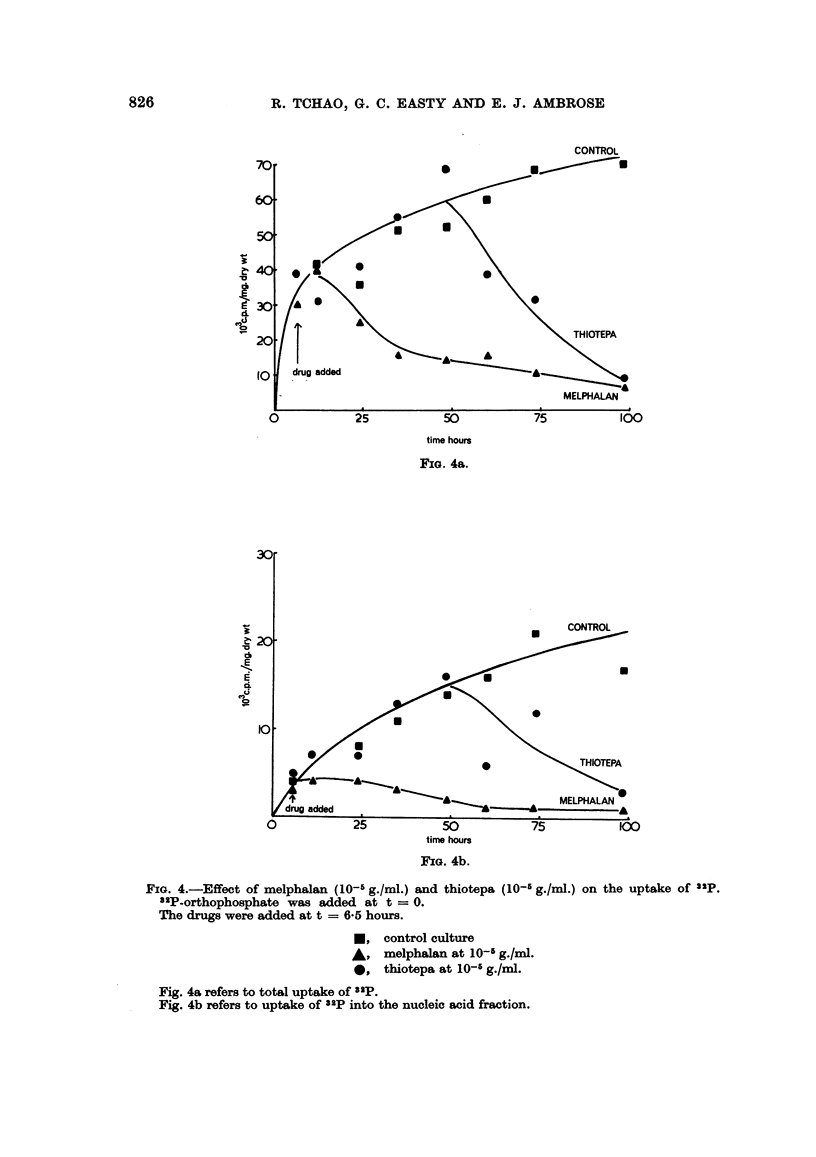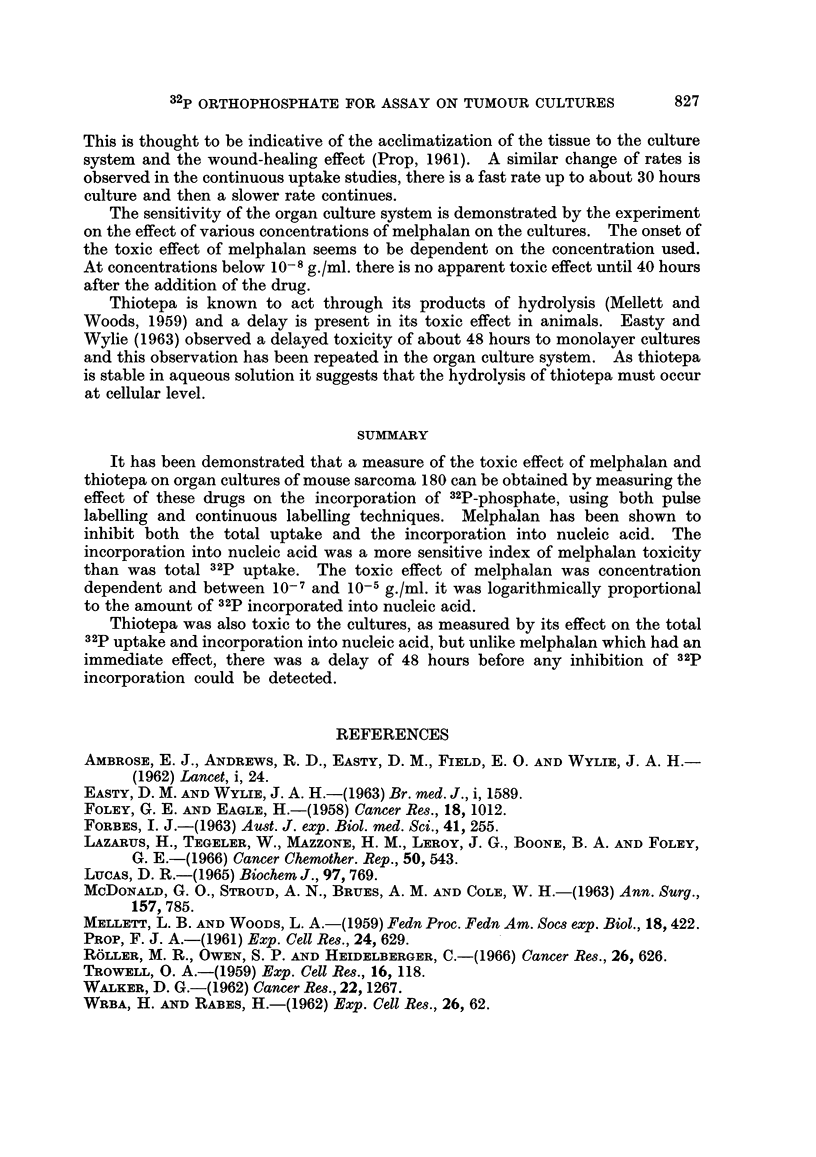# The Use of ^32^P Labelled Orthophosphate for the Assay of Chemotherapeutic Agents on Tumours Maintained in Organ Culture

**Published:** 1967-12

**Authors:** R. Tchao, G. C. Easty, E. J. Ambrose


### THE USE OF $^{32}P$ LABELLED ORTHOPHOSPHATE FOR THE ASSAY OF CHEMOTHERAPEUTIC AGENTS ON TUMOURS MAINTAINED IN ORGAN CULTURE

R. TCHAO, G. C. EASTY AND E. J. AMBROSE

*From the Chester Beatty Research Institute, Institute of Cancer Research: Royal Cancer Hospital, Fulham Road, London S.W.3*

Received for publication July 27, 1967

A NUMBER of systems have been developed for assessing the effects of chemotherapeutic agents on tumour cells *in vitro*. These include monolayer cultures (Foley and Eagle, 1958), explant cultures (Walker, 1962), organ cultures (Röller, Owen and Heidelberger, 1966; Ambrose *et al.*, 1962), and a combination of a variety of *in vitro* techniques (Lazarus *et al.*, 1966). In general, for solid human tumours, organ cultures can be considered to be closer to the *in vivo* situation than monolayer cultures. For example, in monolayer cultures, only a small and not necessarily representative proportion of the original cell population of the tissue gives rise to the final cultures on which the effects of the drugs are examined. In addition, in monolayer cultures the interactions between the cells which may influence the effects of the drugs are greatly reduced.

One obstacle to the routine use of organ cultures compared with monolayers has been the greater difficulty of assessing the effects of drugs. Measurements of cell number and viability are much more difficult than in monolayer culture. Most assessments have been made on the basis of histological examination of sections of the organ cultures, which is extremely difficult to quantitate. The most obvious method of obtaining some quantitative measure of cell viability in this system is to measure the uptake or release of a radioactive label which is involved in an essential metabolic process within the cells. Several methods have been developed for monolayer cultures or cell suspensions. Forbes (1963) has made use of the measurement of the release of $^{32}P$ from cells labelled with $^{32}PO_4$ as an index of cell death, and McDonald *et al.* (1963) have used the uptake of $^{3}H$-thymidine and $^{3}H$-uridine for assessing the effects of cytotoxic drugs on suspensions of Ehrlich ascites tumour cells.

Few attempts have been made to apply such quantitative techniques to organ cultures. Wrba and Rabes (1962) have used $^{32}PO_4$ uptake as an indication of viability of organ cultures of rat liver, and Lucas (1965) has described an elegant, but complex method for measuring the $O_2$ consumption of organ cultures which could be used for examining the effects of chemotherapeutic agents on human tissues *in vitro*.

In the present study, the uptake of radioactive metabolites by organ cultures has been used as an index of the viability of the cultured tissues after treatment with chemotherapeutic agents, and this paper reports the use of $^{32}P$ orthophosphate as the radioactive tracer to test the sensitivity of mouse sarcoma 180 (Crocker) to melphalan and to a lesser extent thiotepa.

### METHODS AND MATERIAL

The organ culture method was modified from Trowell's (1959) grid method· Fragments of mouse Crocker sarcoma 180 1–2 mm$^3$, were placed on lens tissue paper supported by stainless steel mesh grids in disposable plastic petri dishes, each dish containing 5 ml. culture medium. The medium was TC 109 plus 10% horse serum and 10% chick embryo extract. Several dishes were kept in a gassing chamber made from a plastic box with gas taps fitted on the lid. The lid was then sealed to the box with sellotape. Ninety-five per cent $O_2$ and 5% $CO_2$ mixture was used as the gas phase for culture. The boxes containing cultures were gassed for 20–30 minutes at a rate of 1 litre/minute. The cultures were maintained for 3–4 days, during which time the tissue fragments appeared quite healthy as judged by histology.

The tracer $^{32}$P-orthophosphate was used at a final concentration of 20 $\mu$Ci/ml., in the culture medium. In some experiments the $^{32}$P-phosphate was added when the cultures were set up, and in others, as in pulse label experiments, it was added 48 to 72 hours later. The radioactive tracer was not diluted with carrier as the medium contained sufficient " cold " phosphate. For each experiment the results were corrected for the decay of $^{32}$P. The $^{32}$P activity was measured on an end window counter.

The extraction procedure of the tissues was as follows:

i. Saline wash (3 × 2 ml.), residue kept.
ii. Ethyl alcohol wash (2 × 2 ml.). The tissue was then air dried and weighed. $^{32}$P activity of the residue was measured giving total $^{32}$P counts.
iii. Cold TCA (0·5 M) extraction (3 × 2 ml.), residue kept.
iv. Ethyl alcohol wash, followed by lipid extraction. (Ethyl alcohol, chloroform, ether at 60° C.).
v. The residue was counted for $^{32}$P activity.

Preliminary experiments showed that after extraction (iv) further extraction by hot TCA practically removed all $^{32}$P activity, therefore the $^{32}$P activity remaining after extraction procedure iv. gave the activity in the nucleic acid fraction.

For each assessment of $^{32}$P incorporation, several specimens were used, the dry weights of each specimen being between 300–800 $\mu$g.

The chemotherapeutic agents, melphalan and thiotepa, were usually added a few hours after the cultures were set up. Melphalan was dissolved in alc. HCl and propylene glycol. Thiotepa was soluble in water at the concentrations used in the experiments. Usually 0·1 ml. drug solution was added to 5 ml. medium.

### RESULTS

The rate of total and nucleic acid incorporation of $^{32}$P by culture of sarcoma 180 cultured for a period of 4 days in the presence and absence of melphalan is illustrated in Fig. 1a, b. $^{32}$P-phosphate was added to the medium when the cultures were set up and melphalan, at a final concentration of $10^{-5}$ g./ml., was added to some of the culture dishes 18 hours after the cultures were set up. Samples of the cultures were removed at suitable time intervals from dishes with and without melphalan and extracted for total $^{32}$P and $^{32}$P present in the nucleic acid fraction. The effect of melphalan on the total uptake of $^{32}$P-phosphate is shown in Fig. 1a, and the effect on $^{32}$P-phosphate in the nucleic acid fraction in

Fig. 1b. Melphalan inhibited both fractions of $^{32}$P uptake but the incorporation into the nucleic acid fraction was more rapidly inhibited than was the total uptake.

FIG. 1a.

FIG. 1b.

FIG. 1.—Uptake of $^{32}$P by tissues in the presence and absence of melphalan at $10^{-5}$ g./ml. concentration.

Inset. Histogram of $^{32}$P uptake measured by pulse. Shaded histogram refers to cultures in the presence of melphalan.

Fig. 1a refers to total uptake of $^{32}$P.

Fig. 1b refers to uptake of $^{32}$P into nucleic acid fraction.

Some of the cultures were set aside for pulse labelling with $^{32}$P-phosphate for 7 hours at 0, 48 and 72 hours after the beginning of culture. The results are shown for total uptake (inset Fig. 1a) and for nucleic acid incorporation (inset Fig. 1b). The open histograms refer to the uptake by control cultures and the

$^{32}P$ uptake of the melphalan treated cultures is represented by the shaded histograms. As was observed with the continuous labelling technique, the effect of melphalan on the pulse-labelling at 48 and 72 hours was greater when measured by the nucleic acid incorporation of $^{32}P$ than by the total uptake.

In the next experiments, varying concentrations of melphalan were tested against the cultures, the result of which is shown in Fig. 2. Melphalan at concentrations of $10^{-8}$, $10^{-7}$, $10^{-6}$ and $10^{-5}$ g./ml. was added to cultures 6 hours after they were set up and the incorporation of $^{32}P$-phosphate into the nucleic acid fraction measured at intervals up to 48 hours. Melphalan at a final concentration of $10^{-5}$ g./ml. showed an immediate toxic effect on the incorporation of $^{32}P$ into the nucleic acid fraction, the rate decreased within an hour after the addition of melphalan. At $10^{-6}$ g./ml. the decrease in $^{32}P$ uptake was not apparent until 12

FIG. 2.—The uptake of $^{32}P$ into the nucleic acid fraction of tissues cultured in the presence of various concentrations of melphalan.

●, control culture without melphalan
▲, $10^{-8}$ g./ml. melphalan
□, $10^{-7}$ g./ml. melphalan
■, $10^{-6}$ g./ml. melphalan
○, $10^{-5}$ g./ml. melphalan hours later and at $10^{-7}$ and $10^{-8}$ g./ml. the drug was relatively ineffective, although a slight reduction in the incorporation of $^{32}P$ was observed at 30 hours culture.

The dose dependent effect of melphalan was also demonstrated with pulse label experiment. Fig. 3 illustrates the result of the experiment. Melphalan at concentrations of logarithmic dilutions between $10^{-8}$ and $10^{-4}$ g./ml. was added 18 hours after the beginning of the culture and the tracer $^{32}P$ was introduced to the cultures at 23 hours. The incorporation of $^{32}P$ into the nucleic acid fraction was measured after 18 hours. From a plot of the minus log (concentration of drug) versus $^{32}P$ uptake, it can be seen that the effective concentrations of melphalan that can be studied in this system are between $10^{-7}$ g./ml. and $10^{-5}$ g./ml., where a linear relationship between the drug concentration and $^{32}P$ uptake is obtained.

Experiments were also carried out to compare the toxicity of thiotepa with that of melphalan. It was observed that when the two drugs were applied at a concentration of $10^{-5}$ g./ml., melphalan exhibited an immediate effect on the

FIG. 3.—Effect of melphalan at various concentrations on the uptake of $^{32}P$ into the nucleic acid fraction. C refers to concentrations (g./ml.) of melphalan added to the cultures. The dotted line refers to the $^{32}P$ uptake by control cultures without melphalan.

cultures whereas thiotepa showed a delay of 48 hours before any toxic effect on the culture was observed. Fig. 4a and b illustrate the result of the experiment. $^{32}P$-phosphate was added at the beginning of the culture and the drugs were added 6 hours afterwards. The total $^{32}P$ uptake is shown in Fig. 4a, and the uptake into nucleic acid fraction is shown in Fig. 4b. The toxic effect of thiotepa, although delayed for about 48 hours, is finally comparable to the effect of melphalan on these cultures.

### DISCUSSION

This series of experiments showed that the effect of melphalan on organ cultures of solid tumour can be expressed quantitatively by using the uptake of tracers such as $^{32}P$-orthophosphate.

$^{32}P$ incorporation into the nucleic acid fraction is more sensitive than the total uptake to the toxic effect of melphalan. Similar observations were made by McDonald and colleagues (1963) using the pulse label of $H^3$-TdR and $H^3$-UdR. They have shown that the incorporation of tracers into DNA was the most sensitive fraction of the cells. However, a specific label such as $H^3$-TdR measures only the effect of the drug on cells entering into the S-phase, but it has the advantage of reducing the background label.

In our system, both pulse and continuous labelling methods have been used. We have shown that the effect of melphalan on these cultures is more rapidly and clearly shown in the continuous labelling method. In the experiment shown in Fig. 1 the pulse labelling is illustrated in the inset of the figure. At 30 hours after the addition of melphalan, the $^{32}P$ uptake in the treated cultures was 50% of the control, and at 50 hours, 30% of the control. The continuous uptake curve shows that the treated cultures have in fact ceased to incorporate $^{32}P$ shortly after the addition of melphalan, and some $^{32}P$ already incorporated was released from the tissues. Immediately after the addition of the drug the slopes of the curve of $^{32}P$ uptake changed from positive to negative, and continued from then on, whereas the control uptake curve showed always a positive curve.

The pulse label experiments showed that the rate of $^{32}P$ incorporation at the beginning of the culture was always higher than the rate after 48 hours culture.

FIG. 4a.

FIG. 4b.

FIG. 4.—Effect of melphalan ($10^{-5}$ g./ml.) and thiotepa ($10^{-5}$ g./ml.) on the uptake of $^{32}P$.
$^{32}P$-orthophosphate was added at t = 0.
The drugs were added at t = 6·5 hours.

■, control culture
▲, melphalan at $10^{-5}$ g./ml.
●, thiotepa at $10^{-5}$ g./ml.

Fig. 4a refers to total uptake of $^{32}P$.
Fig. 4b refers to uptake of $^{32}P$ into the nucleic acid fraction.

This is thought to be indicative of the acclimatization of the tissue to the culture system and the wound-healing effect (Prop, 1961). A similar change of rates is observed in the continuous uptake studies, there is a fast rate up to about 30 hours culture and then a slower rate continues.

The sensitivity of the organ culture system is demonstrated by the experiment on the effect of various concentrations of melphalan on the cultures. The onset of the toxic effect of melphalan seems to be dependent on the concentration used. At concentrations below $10^{-8}$ g./ml. there is no apparent toxic effect until 40 hours after the addition of the drug.

Thiotepa is known to act through its products of hydrolysis (Mellett and Woods, 1959) and a delay is present in its toxic effect in animals. Easty and Wylie (1963) observed a delayed toxicity of about 48 hours to monolayer cultures and this observation has been repeated in the organ culture system. As thiotepa is stable in aqueous solution it suggests that the hydrolysis of thiotepa must occur at cellular level.

SUMMARY

It has been demonstrated that a measure of the toxic effect of melphalan and thiotepa on organ cultures of mouse sarcoma 180 can be obtained by measuring the effect of these drugs on the incorporation of $^{32}P$-phosphate, using both pulse labelling and continuous labelling techniques. Melphalan has been shown to inhibit both the total uptake and the incorporation into nucleic acid. The incorporation into nucleic acid was a more sensitive index of melphalan toxicity than was total $^{32}P$ uptake. The toxic effect of melphalan was concentration dependent and between $10^{-7}$ and $10^{-5}$ g./ml. it was logarithmically proportional to the amount of $^{32}P$ incorporated into nucleic acid.

Thiotepa was also toxic to the cultures, as measured by its effect on the total $^{32}P$ uptake and incorporation into nucleic acid, but unlike melphalan which had an immediate effect, there was a delay of 48 hours before any inhibition of $^{32}P$ incorporation could be detected.

### REFERENCES

AMBROSE, E. J., ANDREWS, R. D., EASTY, D. M., FIELD, E. O. AND WYLIE, J. A. H.—(1962) *Lancet*, i, 24.
EASTY, D. M. AND WYLIE, J. A. H.—(1963) *Br. med. J.*, i, 1589.
FOLEY, G. E. AND EAGLE, H.—(1958) *Cancer Res.*, **18,** 1012.
FORBES, I. J.—(1963) *Aust. J. exp. Biol. med. Sci.*, **41,** 255.
LAZARUS, H., TEGELER, W., MAZZONE, H. M., LEROY, J. G., BOONE, B. A. AND FOLEY, G. E.—(1966) *Cancer Chemother. Rep.*, **50,** 543.
LUCAS, D. R.—(1965) *Biochem J.*, **97,** 769.
MCDONALD, G. O., STROUD, A. N., BRUES, A. M. AND COLE, W. H.—(1963) *Ann. Surg.*, **157,** 785.
MELLETT, L. B. AND WOODS, L. A.—(1959) *Fedn Proc. Fedn Am. Socs exp. Biol.*, **18,** 422.
PROP, F. J. A.—(1961) *Exp. Cell Res.*, **24,** 629.
RÖLLER, M. R., OWEN, S. P. AND HEIDELBERGER, C.—(1966) *Cancer Res.*, **26,** 626.
TROWELL, O. A.—(1959) *Exp. Cell Res.*, **16,** 118.
WALKER, D. G.—(1962) *Cancer Res.*, **22,** 1267.
WRBA, H. AND RABES, H.—(1962) *Exp. Cell Res.*, **26,** 62.